# Replication Study for the Association of 9 East Asian GWAS-Derived Loci with Susceptibility to Type 2 Diabetes in a Japanese Population

**DOI:** 10.1371/journal.pone.0076317

**Published:** 2013-09-25

**Authors:** Kensuke Sakai, Minako Imamura, Yasushi Tanaka, Minoru Iwata, Hiroshi Hirose, Kohei Kaku, Hiroshi Maegawa, Hirotaka Watada, Kazuyuki Tobe, Atsunori Kashiwagi, Ryuzo Kawamori, Shiro Maeda

**Affiliations:** 1 Laboratory for Endocrinology, Metabolism and Kidney Diseases, RIKEN Center for Integrative Medical Sciences, Yokohama, Japan; 2 Department of Internal Medicine, Division of Metabolism and Endocrinology, St. Marianna University School of Medicine, Kawasaki, Japan; 3 First Department of Internal Medicine, University of Toyama, Toyama, Japan; 4 Health Center, Keio University School of Medicine, Tokyo, Japan; 5 Division of Diabetes, Endocrinology and Metabolism, Department of Internal Medicine, Kawasaki medical school, Kurashiki, Japan; 6 Department of Medicine, Shiga University of Medical Science, Otsu, Japan; 7 Department of Metabolism and Endocrinology, Juntendo University Graduate School of Medicine, Tokyo, Japan; 8 Sportology Center, Juntendo University Graduate School of Medicine, Tokyo, Japan; University of Siena, Italy

## Abstract

**Aims:**

East Asian genome-wide association studies (GWAS) for type 2 diabetes identified 8 loci with genome-wide significance, and 2 loci with a borderline association. However, the associations of these loci except *MAEA* locus with type 2 diabetes have not been evaluated in independent East Asian cohorts. We performed a replication study to investigate the association of these susceptibility loci with type 2 diabetes in an independent Japanese population.

**Methods:**

We genotyped 7,379 Japanese participants (5,315 type 2 diabetes and 2,064 controls) for each of the 9 single nucleotide polymorphisms (SNPs), rs7041847 in *GLIS3*, rs6017317 in *FITM2*−*R3HDML*−*HNF4A*, rs6467136 near *GCCI*−*PAX4*, rs831571 near *PSMD6*, rs9470794 in *ZFAND3*, rs3786897 in *PEPD*, rs1535500 in *KCNK16*, rs16955379 in *CMIP*, and rs17797882 near *WWOX*. Because the sample size in this study was not sufficient to replicate single SNP associations, we constructed a genetic risk score (GRS) by summing a number of risk alleles of the 9 SNPs, and examined the association of the GRS with type 2 diabetes using logistic regression analysis.

**Results:**

With the exception of rs1535500 in *KCNK16*, all SNPs had the same direction of effect (odds ratio [OR]>1.0) as in the original reports. The GRS constructed from the 9 SNPs was significantly associated with type 2 diabetes in the Japanese population (*p* = 4.0 × 10^-4^, OR = 1.05, 95% confidence interval: 1.02–1.09). In quantitative trait analyses, rs16955379 in *CMIP* was nominally associated with a decreased homeostasis model assessment of β-cell function and with increased fasting plasma glucose, but neither the individual SNPs nor the GRS showed a significant association with the glycemic traits.

**Conclusions:**

These results indicate that 9 loci that were identified in the East Asian GWAS meta-analysis have a significant effect on the susceptibility to type 2 diabetes in the Japanese population.

## Introduction

Diabetes mellitus affects more than 300 million individuals worldwide, and its prevalence is progressively increasing, particularly in the East Asia, South Asia and Western Pacific regions [1]. Although the precise mechanisms underlying the development and progression of type 2 diabetes have not been elucidated, previous epidemiological studies have linked diverse factors, such as obesity, hypertension and dyslipidemia, with the risk of type 2 diabetes [2]. Additionally, genetic factors are thought to play an important role in the pathogenesis of type 2 diabetes [3]. Genetic studies of type 2 diabetes have made rapid progress through genome-wide association studies (GWAS) [4,5], and the number of susceptibility loci identified for type 2 diabetes has been increased through GWAS to nearly 70 [6–25]. In 2012, an East Asian GWAS meta-analysis identified 8 susceptibility loci for type 2 diabetes [23], namely rs6815464 in *MAEA*, rs7041847 in *GLIS3*, rs6017317 in *FITM2*−*R3HDML*−*HNF4A* locus, rs6467136 near *GCC1*-*PAX4* locus, rs831571 near *PSMD6*, rs9470794 in *ZFAND3*, rs3786897 in *PEPD*, and rs1535500 in *KCNK16*, along with 2 additional loci with borderline association (*p* < 10^-6^), namely rs16955379 in *CMIP* and rs17797882 near *WWOX*. Of these, the association of rs6815464 in *MAEA* with type 2 diabetes has been already evaluated and confirmed in an independent Japanese population [25], but the associations of the remaining 9 loci have not been evaluated in an independent East Asian cohort. In the East Asian GWAS meta-analysis reported by Cho et al. [23], over 50,000 individuals were analyzed, and sample sizes that are considered large by conventional standards (~ several thousands) may be underpowered to conduct individual single nucleotide polymorphism (SNP) analyses. It was also shown that a genetic risk score (GRS) constructed by summing the number of risk alleles for multiple loci had a much stronger effect on disease susceptibility than individual SNPs; thus, the GRS is considered to be a useful and tractable measure to evaluate the effect of multiple candidate loci of interest [26–28]. In this study, to evaluate the contribution of these 9 loci identified in East Asian GWAS meta-analysis to conferring susceptibility to type 2 diabetes in an independent population, we performed a replication study for the association of these 9 susceptibility loci with type 2 diabetes in an independent Japanese population using the GRS that was constructed from the 9 loci.

## Materials and Methods

### Ethics Statements

All participants agreed to the protocol of this study and provided written informed consent before enrolling. DNA samples were obtained from the peripheral blood of each participant. The study protocol conformed to the provisions of the Declaration of Helsinki, and was approved by the ethics committees of RIKEN Yokohama Institute and each of the participating institutes, i.e., Shiga University of Medical Science, Kawasaki Medical School, St. Marianna University, Juntendo University, the University of Toyama, and Keio University.

### Participants and DNA Preparation

We enrolled 5,315 type 2 diabetes patients who regularly visited the outpatient clinics of Shiga University of Medical Science, Kawasaki Medical School, St. Marianna University, Juntendo University, and the University of Toyama or who were registered in BioBank Japan [12]. Diabetes mellitus was diagnosed according to the World Health Organization (WHO) criteria [29], and type 2 diabetes was defined by a gradual adult onset. Patients who tested positive for antibodies to glutamic acid decarboxylase or who were diagnosed with mitochondrial disease or maturity-onset diabetes of the young were excluded from the present study. We also recruited 2,064 controls, who underwent annual health check-ups at Keio University, St. Marianna University, or Toyama University Hospital.

### Single nucleotide polymorphism (SNP) Genotyping

We first selected the following 8 SNPs identified by GWAS in individuals of East Asian ancestry and were reported by Cho et al. in 2012 [23], rs6815464 in *MAEA*, rs7041847 in *GLIS3*, rs6017317 in *FITM2*−*R3HDML*−*HNF4A* locus, rs6467136 near *GCC1*-*PAX4* locus, rs831571 near *PSMD6*, rs9470794 in *ZFAND3*, rs3786897 in *PEPD*, and rs1535500 in *KCNK16*. Because the association of rs6815464 in *MAEA* with type 2 diabetes was already evaluated, and confirmed in the present Japanese population [25], we excluded this SNP from the present study. We also examined 2 additional SNP loci, that showed a borderline association (*p* < 10^-6^) in the East Asian GWAS meta-analysis, rs16955379 in *CMIP* and rs17797882 near *WWOX*.

Genotyping was performed using the multiplex-polymerase chain reaction (PCR) invader assay as previously described [30]. The success rates of the assay were > 95% for each SNP, and the concordance rates in the duplicate samples were ≥ 99.8%.

### Statistical Analysis

We performed Hardy-Weinberg equilibrium (HWE) tests according to the method described by Nielsen et al [31]. The SNP genotypes were scored using an additive model (0, 1, and 2 for homozygous for the non-effect allele, heterozygous, and homozygous for the effect allele, respectively). We calculated a GRS by counting the number of risk alleles of the 9 SNPs in each individual. The association of the GRS or each SNP with type 2 diabetes was evaluated by a logistic regression analysis with or without adjustment for age, sex, and body mass index (BMI). The association of the GRS or each SNP with quantitative traits, including fasting plasma glucose (FPG), the homeostasis model assessment of β-cell function (HOMA-β), and the HOMA of insulin resistance (HOMA-IR) [32,33] was evaluated by multiple linear regression analysis. The values of these traits in the present Japanese population showed a skewed distribution; therefore, we used log-transformed values for the analyses.

Statistical analyses were performed using StatView software (SAS Institute, Cary, NC, USA). The level of significance was determined by the Bonferroni correction for multiple tests, and a *p* value of <0.0055 (0.05 divided by 9) was considered to be statistically significant.

## Results

The clinical characteristics of the participants are shown in Table 1. The male ratio, age and BMI in the type 2 diabetes cases were significantly higher than those in the controls. The genotype distributions for all 9 SNPs did not deviate from HWE proportions, except rs7041847 in the type 2 diabetes cases (*p* = 0.015, Table 2). Since our samples were collected at 7 distinct sites for patients with type 2 diabetes and at 3 sites for the control individuals, we compared the genotype distributions of the 9 SNPs among the different collection sites. The results indicated that the genotype distributions of each SNP were not significantly different among the 7 collection sites for type 2 diabetes or among the 3 collection sites for the controls with 1 exception; the genotype distribution of rs16955379 was slightly different among the 7 type 2 diabetes collection sites (*p* = 0.0339, Table S1), although the difference was not statistically significant after correction for multiple testing error.

**Table 1:** Clinical characteristics of participants.

	Sample size (case/control)	Type 2 diabetes	Control	*p*
n		5,315	2,064	
Sex (male:female)	5,315/2,064	3,249 : 2,066	982 : 1,082	< 0.0001^b^
Age (year)^a^	5,278/2,058	63.0 ± 11.5	49.8 ± 17.0	< 0.0001^c^
BMI (kg/m^2^)^a^	4,951/2,018	24.2 ± 4.1	22.4 ± 3.1	< 0.0001^c^
HbA1c (%)^a^	4,730/1,386	7.9 ± 2.1	5.4 ± 0.3	< 0.0001^c^
FPG (mmol/L)^a^	2,249/1,320	8.4 ± 3.0	5.3 ± 0.6	< 0.0001^c^
Diabetes duration (year)^a^	3,474/-	13.7 ± 9.7		

^a^ Data are mean ± SD

^b^ Chi-square test

^c^ Mann-Whitney test

BMI: body mass index, HbA1c: Glycated hemoglobin, FPG: fasting plasma glucose

**Table 2:** Genotype distributions of 9 single nucleotide polymorphisms (SNPs) in case and control groups.

SNP	Gene	Allele1/Allele2^a^	Allele 11/12/22		*p* for HWE test	
			Type 2 diabetes	Control	Type 2 diabetes	Control
rs7041847	*GLIS3*	**A**/G	1,256/2,477/1,399	464/982/536	0.0151	0.7293
rs6017317	*FITM2-R3HDML-HNF4A*	**G**/T	1,646/2,639/983	613/1,038/393	0.1908	0.2128
rs6467136	*GCC1-PAX4*	**G**/A	3,144/1,807/279	1,196/727/118	0.3578	0.5884
rs831571	*PSMD6*	**C**/T	2,224/2,372/594	824/949/254	0.3100	0.4505
rs9470794	*ZFAND3*	T/**C**	3,338/1,711/227	1,332/655/68	0.6792	0.2493
rs3786897	*PEPD*	**A**/G	1,733/2,518/1,013	606/1,048/391	0.0689	0.0998
rs1535500	*KCNK16*	G/**T**	2,104/2,400/747	821/945/283	0.1380	0.6738
rs16955379	*CMIP*	C/**T**	2,944/2,012/322	1,181/729/137	0.3749	0.0912
rs17797882	*WWOX*	**C**/T	3,349/1,709/199	1,289/664/92	0.3159	0.5821

^a^ The reported risk allele is indicated in bold

HWE: Hardy-Weinberg equilibrium

The results of the association study for each SNP with type 2 diabetes in the present Japanese population are shown in Table 3. All SNPs showed the same direction of effect (odds ratio [OR] >1.0) as in the original reports [23], except rs1535500 in *KCNK16* (OR = 1.00 in the unadjusted data, OR = 0.99 in the data adjusted for age, sex and BMI). Two SNPs, rs831571 in *PSMD6* and rs3786897 in *PEPD* were nominally associated with type 2 diabetes (rs831571: *p* = 0.0135, OR = 1.11, 95% confidence interval [CI] 1.02–1.22; rs3786897: *p* = 0.0407, OR = 1.09, 95% CI 1.004–1.18, adjusted for age, sex, and BMI); however, these associations were not significant after Bonferroni’s correction (*p* > 0.0055). The remaining 7 SNPs were not associated with type 2 diabetes in the present Japanese population (*p*≥0.05). Although we did not observe a significant association of 9 SNPs with type 2 diabetes by themselves, a GRS constructed from the 9 SNPs was significantly associated with type 2 diabetes in the present Japanese population (*p* = 0.002, per allele OR = 1.04, 95% CI 1.01–1.07, unadjusted data, p = 0.0004, per allele OR = 1.05, 95% CI 1.02–1.09, adjusted for age, sex and BMI).

**Table 3:** Association of 9 SNPs with type 2 diabetes in the Japanese population.

SNP	Gene		Risk Allele^a^	RAF^b^ (case/control)	Unadjusted		Adjusted^c^	
					*p* value	OR (95%CI)	*p* value	OR (95%CI)
rs7041847	*GLIS3*		A	0.486/0.481	0.6550	1.01 (0.94–1.09)	0.3889	1.03 (0.95–1.12)
rs6017317	*FITM2-R3HDML-HNF4A*		G	0.562/0.553	0.3141	1.03 (0.96–1.11)	0.1554	1.06 (0.97–1.15)
rs6467136	*GCC1-PAX4*		G	0.773/0.764	0.2084	1.05 (0.97–1.15)	0.1332	1.07 (0.97–1.19)
rs831571	*PSMD6*		C	0.657/0.640	0.0606	1.07 (0.99–1.16)	0.0135	1.11 (1.02–1.22)
rs9470794	*ZFAND3*		C	0.205/0.192	0.0844	1.08 (0.98–1.18)	0.1513	1.08 (0.97–1.20)
rs3786897	*PEPD*		A	0.568/0.552	0.0846	1.06 (0.99–1.14)	0.0407	1.09 (1.004–1.18)
rs1535500	*KCNK16*		T	0.370/0.368	0.8175	1.00 (0.93–1.12)	0.8599	0.99 (0.91–1.08)
rs16955379	*CMIP*		T	0.251/0.244	0.4067	1.03 (0.95–1.12)	0.1199	1.08 (0.98–1.19)
rs17797882	*WWOX*		C	0.799/0.792	0.3355	1.04 (0.95–1.14)	0.7521	1.01 (0.91–1.12)
GRS^d^					0.0020	1.04 (1.01–1.07)	0.0004	1.05 (1.02–1.09)

The results of logistic regression analyses are shown

a The risk allele reported in the previous report

b Risk allele frequency

c Adjusted for age, sex and log-transformed BMI

d The genetic risk score (GRS) was calculated according to the number of risk alleles by counting the 9 East Asian genome-wide association study derived SNPs; Individuals who had complete genotype data for the 9 SNPs were used for the analysis (n = 6,819)

We next examined the associations between these SNPs and quantitative traits related to glucose metabolism, such as HOMA-IR, HOMA-β, and FPG, using the control individuals (Table 4). Rs16955379-T in *CMIP* had a nominal effect on decreasing HOMA-β (*p* = 0.0442, β= -0.055, se = 0.027) or on increasing FPG (*p* = 0.0271, β= 0.009, se = 0.004). However, neither the individual SNPs nor the GRS was significantly associated with these quantitative traits after Bonferroni’s correction in the present Japanese population.

**Table 4:** Association of 9 SNPs with quantitative traits related to glucose metabolism in controls.

SNP	Gene	Risk Allele^a^	HOMA-IR^b^ (n=893)		HOMA-β^b^ (n=893)		FPG^b^ (n=1,325)	
			Effect (SE)	*p* value	Effect (SE)	*p* value	Effect (SE)	*p* value
rs7041847	*GLIS3*	A	-0.011 (0.023)	0.6441	-0.015 (0.024)	0.5429	0.003 (0.004)	0.3839
rs6017317	*FITM2-R3HDML-HNF4A*	G	-0.015 (0.023)	0.5123	0.038 (0.024)	0.1184	-0.006 (0.004)	0.1052
rs6467136	*GCC1-PAX4*	G	0.008 (0.027)	0.7672	0.028 (0.028)	0.3094	-0.001 (0.004)	0.7666
rs831571	*PSMD6*	C	-0.006 (0.024)	0.8066	0.029 (0.025)	0.2384	-0.006 (0.004)	0.0963
rs9470794	*ZFAND3*	C	-0.027 (0.029)	0.3613	-0.011 (0.031)	0.7129	-0.004 (0.005)	0.4310
rs3786897	*PEPD*	A	0.040 (0.023)	0.0894	0.014 (0.025)	0.5728	0.005 (0.004)	0.1517
rs1535500	*KCNK16*	T	-0.007 (0.023)	0.7586	-0.003 (0.024)	0.9032	0.003 (0.004)	0.3892
rs16955379	*CMIP*	T	-0.028 (0.026)	0.2870	-0.055 (0.027)	0.0442	0.009 (0.004)	0.0271
rs17797882	*WWOX*	C	0.0015 (0.028)	0.5941	0.003 (0.030)	0.9064	0.002 (0.004)	0.5919
GRS^c^			-0.004 (0.009)	0.6271	0.006 (0.009)	0.5398	0.0003 (0.001)	0.8405

The results of linear regression analysis after adjusting for age, sex and log-transformed BMI are presented.

^a^ The risk allele for type 2 diabetes reported in the previous report

^b^ Values are log-transformed before the analysis

^c^ The GRS was calculated according to the number of risk alleles of the 9 SNPs, and the individuals who had complete genotype data for the 9 SNPs were used for the analysis (n= 844 for HOMA-IR and HOMA-β, n=1,181 for FPG)

HOMA-IR: homeostasis model assessment of insulin resistance, HOMA-β: HOMA of β cell function, FPG: fasting plasma glucose

## Discussion

In this study, we examined the association of 9 SNP loci derived from an East Asian GWAS meta-analysis with type 2 diabetes in an independent Japanese population, and found that a GRS that was constructed with the 9 SNPs was significantly associated with type 2 diabetes in the present Japanese population.

To date, nearly 70 susceptibility loci have been identified for type 2 diabetes [6–25], through GWAS, and a few plausible candidates were reported through candidate gene approaches [34,35]. Of these loci, several have been shown to be associated with type 2 diabetes in different ethnic groups, including the Japanese [28,36–41]. In 2012, a GWAS meta-analysis that was conducted by the Asian Genetic Epidemiology Network Consortium for type 2 diabetes (AGEN-T2D) identified 8 loci that were associated with susceptibility to type 2 diabetes at genome-wide significance levels, and 2 additional loci with borderline association (*p* < 10^-6^) [23]. Although these loci are considered to be important suceptibility loci for type 2 diabetes in East Asian populations, there are some differences in genetic background among different East Asian populations [42]. The *MAEA* locus was previously shown to be associated with type 2 diabetes in the present Japanese sample [25], but the remaining 9 loci have not been examined in an independent East Asian cohort. Therefore, the associations of these loci with type 2 diabetes need to be evaluated in independent ethnic groups to know the contribution of each locus to susceptibility to the disease in individual ethnic groups.

In the present Japanese population, we did not observe a significant association of all the 9 SNPs with type 2 diabetes. The accuracy of the genotyping was well validated, and the genotype distribution for each SNP in the controls did not deviate from HWE proportions, indicating that techinical factors are not likely to be a principal cause of the discrepancy between the present study and the original study. Although we collected samples for case and control individuals from several different Japanese institutes, the genotype distributions for the individual SNPs were not significantly different among the different collection sites (Table S1); therefore, we considered that our finding was not affected by sample heterogeneity.

Because the control individuals in this study were younger than the type 2 diabetes patients, the control group might include several individuals who would develop the disease later, and the possibility of type 2 error may be increased, although the results were not affected by adjusting for age. We evaluated the association of the 9 SNPs with type 2 diabetes using the older control individuals (age ≥ 40, ≥ 50 or ≥ 60). The results indicated that the effect sizes of individual SNPs were almost the same in the analysis using older controls as those in the original findings (Table S2). Most SNPs showed the same direction of effect as those in the original reports, except rs1535500 in *KCNK16* and the estimated power of the present study to replicate an original association for the individual SNPs is between 42% and 79% (Table S3); therefore, insufficient study power is likely to be a principal cause for the discrepancy between this Japanese study and the original East Asian GWAS meta-analysis. Since a GRS constructed by summing the number of risk alleles for multiple loci was shown to be useful and tractable measure to evaluate the effect of multiple candidate loci of interest [26–28], we examined the combined effect of the 9 SNPs on conferring susceptibility to type 2 diabetes, and found that the GRS constructed using the 9 SNPs was significantly associated with type 2 diabetes; therefore, we suggest that the 9 SNPs are important susceptibility loci for type 2 diabetes also in the Japanese population, and the construction of a GRS is considered to be a useful and alternative approach to evaluate the effect of multiple candidate loci in a underpowered sample for single SNP analyses, like the present study. However, since the association did not attain a genome-wide significance level, the contribution of the 9 loci to susceptibility to Japanese type 2 diabetes needs to be evaluated further in larger Japanese cohorts.

The mechanisms of how these loci contribute to susceptibility to type 2 diabetes are still unknown. The analyses of quantitative traits related to glucose metabolism, such as HOMA-β, HOMA-IR and FPG, revealed that rs16955379 in *CMIP* had a nominal effect on decreased HOMA-β and on increased FPG, but neither individual SNPs nor the GRS were significantly associated with these quantitative traits, probably because of insufficient study power (Table S4). Further study is required to elucidate the precise mechanisms how these loci contribute to conferring susceptibility to type 2 diabetes.

In conclusion, we examined the association of 9 SNPs identified in an East Asian GWAS meta-analysis with type 2 diabetes in an independent Japanese population. Our results indicate that the 9 East Asian GWAS-derived loci have a significant effect on conferring susceptibility to type 2 diabetes in the Japanese population.

## Supporting Information

Table S1 **Comparison of risk allele frequencies among individual areas for sample collection.** ^a^collection 1 (BioBank Japan1), collection 2 (Kawasaki Medical University), collection 3 (BioBank Japan2), collection 4 (Juntendo University), collection 5 (Case; Shiga University of Medical Science, Control; Keio University), collection 6 (St. Mariannna University School of Medicine), collection 7 (Toyama University). ^b^Chi square test.
(DOCX)

Table S2 **Association study of 9 SNPs with type 2 diabetes using older control (age ≥ 40, n=1,424, age ≥ 50, n=1,057, age ≥ 60n=622) and all cases (n=5,315).** Results of logistic regression analysis are shown. ^a^risk allele reported in the previous reports. ^b^adjusted for age, sex and log-transformed BMI.
(DOCX)

Table S3 Power estimation for each SNP locus in the present study. ^a^Risk allele for type 2 diabetes reported in the previous reports. ^b^Power estimation was performed using CaTS power calculator, CaTS: http://www.sph.umich.edu/csg/abecasis/CaTS/). The prevalence of type 2 diabetes is assumed to be 10%, α = 0.05. ^c^Required sample size estimation was performed using the Quanto software package (Version 1.2.4, http://hydra.usc.edu/gxe/).
(DOCX)

Table S4 Estimation of statistical power for the present study to detect associations of 9 SNPs with quantitative metabolic traits. Power calculations were carried out using the Quanto software package (Version 1.2.4, http://hydra.usc.edu/gxe/). ^a^values are log-transformed for the analysis.
(DOCX)
