# Supplementary material for: Replication Study for the Association of 9 East Asian GWAS-Derived Loci with Susceptibility to Type 2 Diabetes in a Japanese Population
Source: PLoS One. 2013 Sep 25;8(9):e76317. doi: 10.1371/journal.pone.0076317 (PMC3783369; doi:10.1371/journal.pone.0076317)
Supplement: Table S1 — Comparison of risk allele frequencies among individual areas for sample collection. acollection 1 (BioBank Japan1), collection 2 (Kawasaki Medical University), collection 3 (BioBank Japan2), collection 4 (Juntendo University), collection 5 (Case; Shiga University of Medical Science, Control; Keio University), collection 6 (St. Mariannna University School of Medicine), collection 7 (Toyama University). bChi square test. (DOCX) [file pone.0076317.s001.docx]

**Table S1** Comparison of risk allele frequencies among individual areas for sample collection

|  |  | Risk allele frequencies | | | | | | |  |
| --- | --- | --- | --- | --- | --- | --- | --- | --- | --- |
| SNP | collection^a^ | 1 | 2 | 3 | 4 | 5 | 6 | 7 | *p*^b^ |
| rs7041847 | case | 0.486 | 0.485 | 0.478 | 0.481 | 0.498 | 0.514 | 0.476 | 0.9249 |
|  | control |  |  |  |  | 0.473 | 0.493 | 0.480 | 0.4677 |
| rs6017317 | case | 0.564 | 0.561 | 0.566 | 0.540 | 0.573 | 0.566 | 0.575 | 0.1795 |
|  | control |  |  |  |  | 0.573 | 0.550 | 0.540 | 0.0562 |
| rs6467136 | case | 0.776 | 0.761 | 0.776 | 0.770 | 0.776 | 0.777 | 0.775 | 0.8064 |
|  | control |  |  |  |  | 0.764 | 0.764 | 0.764 | 0.9607 |
| rs831571 | case | 0.644 | 0.660 | 0.670 | 0.661 | 0.643 | 0.669 | 0.673 | 0.1914 |
|  | control |  |  |  |  | 0.651 | 0.644 | 0.629 | 0.6561 |
| rs9470794 | case | 0.201 | 0.230 | 0.191 | 0.203 | 0.209 | 0.205 | 0.216 | 0.6194 |
|  | control |  |  |  |  | 0.194 | 0.186 | 0.196 | 0.3822 |
| rs3786897 | case | 0.567 | 0.544 | 0.545 | 0.575 | 0.601 | 0.561 | 0.579 | 0.3439 |
|  | control |  |  |  |  | 0.565 | 0.536 | 0.556 | 0.5738 |
| rs1535500 | case | 0.370 | 0.358 | 0.375 | 0.360 | 0.371 | 0.367 | 0.390 | 0.7515 |
|  | control |  |  |  |  | 0.352 | 0.362 | 0.389 | 0.1488 |
| rs16955379 | case | 0.242 | 0.226 | 0.252 | 0.268 | 0.240 | 0.262 | 0.271 | 0.0339 |
|  | control |  |  |  |  | 0.246 | 0.248 | 0.241 | 0.7545 |
| rs17797882 | case | 0.805 | 0.776 | 0.800 | 0.798 | 0.802 | 0.789 | 0.805 | 0.7951 |
|  | control |  |  |  |  | 0.783 | 0.776 | 0.815 | 0.1125 |

^a^collection 1 (BioBank Japan1), collection 2 (Kawasaki Medical University), collection 3 (BioBank Japan2), collection 4 (Juntendo University), collection 5 (Case; Shiga University of Medical Science, Control; Keio University), collection 6 (St. Mariannna University School of Medicine), collection 7 (Toyama University)

^b^Chi square test
