# Supplementary material for: Replication Study for the Association of 9 East Asian GWAS-Derived Loci with Susceptibility to Type 2 Diabetes in a Japanese Population
Source: PLoS One. 2013 Sep 25;8(9):e76317. doi: 10.1371/journal.pone.0076317 (PMC3783369; doi:10.1371/journal.pone.0076317)
Supplement: Table S2 — Association study of 9 SNPs with type 2 diabetes using older control (age ≥ 40, n=1,424, age ≥ 50, n=1,057, age ≥ 60n=622) and all cases (n=5,315). Results of logistic regression analysis are shown. arisk allele reported in the previous reports. badjusted for age, sex and log-transformed BMI. (DOCX) [file pone.0076317.s002.docx]

**Table S2** Association study of 9 SNPs with type 2 diabetes using older control (age ≥ 40, n=1,424, age ≥ 50, n=1,057, age ≥ 60 n=622) and all cases (n=5,315)

| SNP | Gene | Risk Allele^a^ | Control | Unadjusted | | Adjusted^b^ | |
| --- | --- | --- | --- | --- | --- | --- | --- |
|  |  |  |  | *p* value | OR (95%CI) | *p* value | OR (95%CI) |
| rs7041847 | *GLIS3* | A | age ≥ 40 | 0.6639 | 1.02 (0.94−1.11) | 0.5057 | 1.03 (0.94−1.13) |
|  |  |  | age ≥ 50 | 0.9089 | 0.99 (0.90−1.09) | 0.9128 | 0.99 (0.90−1.10) |
|  |  |  | age ≥ 60 | 0.6500 | 1.03 (0.91−1.16) | 0.8745 | 1.01 (0.89−1.14) |
| rs6017317 | *FITM2-*  *R3HDML-*  *HNF4A* | G | age ≥ 40 | 0.2358 | 1.05 (0.97−1.15) | 0.1027 | 1.08 (0.99−1.18) |
|  |  |  | age ≥ 50 | 0.0245 | 1.12 (1.01−1.23) | 0.0116 | 1.13 (1.03−1.25) |
|  |  |  | age ≥ 60 | 0.3150 | 1.06 (0.94−1.20) | 0.1595 | 1.09 (0.97−1.24) |
| rs6467136 | *GCC1-PAX4* | G | age ≥ 40 | 0.3324 | 1.05 (0.95−1.16) | 0.1853 | 1.07 (0.97−1.19) |
|  |  |  | age ≥ 50 | 0.2202 | 1.07 (0.96−1.20) | 0.1166 | 1.10 (0.98−1.23) |
|  |  |  | age ≥ 60 | 0.5917 | 1.04 (0.90−1.19) | 0.3289 | 1.08 (0.93−1.25) |
| rs831571 | *PSMD6* | C | age ≥ 40 | 0.0173 | 1.11 (1.02−1.22) | 0.008 | 1.13 (1.03−1.24) |
|  |  |  | age ≥ 50 | 0.0555 | 1.10 (1.00−1.22) | 0.0212 | 1.13 (1.02−1.25) |
|  |  |  | age ≥ 60 | 0.2433 | 1.08 (0.95−1.22) | 0.1600 | 1.10 (0.96−1.25) |
| rs9470794 | *ZFAND3* | C | age ≥ 40 | 0.0647 | 1.11 (0.99−1.23) | 0.1015 | 1.10 (0.98−1.23) |
|  |  |  | age ≥ 50 | 0.0518 | 1.13 (1.00−1.27) | 0.0731 | 1.12 (0.99−1.27) |
|  |  |  | age ≥ 60 | 0.1896 | 1.11 (0.95−1.29) | 0.2855 | 1.09 (0.93−1.27) |
| rs3786897 | *PEPD* | A | age ≥ 40 | 0.4695 | 1.03 (0.95−1.12) | 0.1776 | 1.06 (0.97−1.16) |
|  |  |  | age ≥ 50 | 0.6858 | 1.02 (0.93−1.12) | 0.6235 | 1.02 (0.93−1.13) |
|  |  |  | age ≥ 60 | 0.2169 | 1.08 (0.96−1.21) | 0.4642 | 1.05 (0.93−1.18) |
| rs1535500 | *KCNK16* | T | age ≥ 40 | 0.3206 | 0.96 (0.88−1.04) | 0.5339 | 0.97 (0.89−1.06) |
|  |  |  | age ≥ 50 | 0.6594 | 0.98 (0.89−1.08) | 0.9515 | 1.00 (0.90−1.10) |
|  |  |  | age ≥ 60 | 0.3709 | 0.95 (0.84−1.07) | 0.7228 | 0.98 (0.86−1.11) |
| rs16955379 | *CMIP* | T | age ≥ 40 | 0.3477 | 1.05 (0.95−1.16) | 0.1627 | 1.08 (0.97−1.19) |
|  |  |  | age ≥ 50 | 0.6650 | 1.02 (0.92−1.14) | 0.4974 | 1.04 (0.93−1.16) |
|  |  |  | age ≥ 60 | 0.5130 | 1.05 (0.91−1.20) | 0.5818 | 1.04 (0.90−1.20) |
| rs17797882 | *WWOX* | C | age ≥ 40 | 0.8903 | 0.99 (0.89−1.10) | 0.9907 | 1.00 (0.89−1.12) |
|  |  |  | age ≥ 50 | 0.3078 | 0.94 (0.83−1.06) | 0.4617 | 0.95 (0.84−1.08) |
|  |  |  | age ≥ 60 | 0.1256 | 0.89 (0.76−1.03) | 0.1203 | 0.88 (0.75−1.03) |
| GRS |  |  | age ≥ 40 | 0.0290 | 1.04 (1.00−1.07) | 0.0022 | 1.05 (1.02−1.09) |
|  |  |  | age ≥ 50 | 0.0410 | 1.04 (1.00−1.08) | 0.0079 | 1.05 (1.01−1.09) |
|  |  |  | age ≥ 60 | 0.2220 | 1.03 (0.98−1.07) | 0.1670 | 1.03 (0.99−1.08) |

Results of logistic regression analysis are shown

^a^risk allele reported in the previous reports

^b^adjusted for age,sex and log-transformed BMI.
