# Supplementary material for: Replication Study for the Association of 9 East Asian GWAS-Derived Loci with Susceptibility to Type 2 Diabetes in a Japanese Population
Source: PLoS One. 2013 Sep 25;8(9):e76317. doi: 10.1371/journal.pone.0076317 (PMC3783369; doi:10.1371/journal.pone.0076317)
Supplement: Table S3 — aRisk allele for type 2 diabetes reported in the previous reports. bPower estimation was performed using CaTS power calculator, CaTS: http://www.sph.umich.edu/csg/abecasis/CaTS/). The prevalence of type 2 diabetes is assumed to be 10%, α = 0.05. cRequired sample size estimation was performed using the Quanto software package (Version 1.2.4, http://hydra.usc.edu/gxe/). (DOCX) [file pone.0076317.s003.docx]

**Table S3** Power estimation for each SNP locus in the present study

| SNP | Gene | Risk Allele^a^ | RAF | Reported OR | Power^b^ | Sample size for 80% power^c^ | | | |  |
| --- | --- | --- | --- | --- | --- | --- | --- | --- | --- | --- |
|  |  |  |  |  |  | α=0.05 | α=0.0055 | α=5×10^-8^ |  |  |
| rs7041847 | *GLIS3* | A | 0.481 | 1.10 | 79% | 8984 | 14932 | 45326 | | |
| rs6017317 | *FITM2-R3HDML-HNF4A* | G | 0.553 | 1.09 | 69% | 13575 | 22563 | 68489 | | |
| rs6467136 | *GCC1-PAX4* | G | 0.764 | 1.11 | 68% | 28714 | 47727 | 144872 | | |
| rs831571 | *PSMD6* | C | 0.640 | 1.09 | 65% | 19354 | 32169 | 97647 | | |
| rs9470794 | *ZFAND3* | C | 0.192 | 1.12 | 75% | 5308 | 8823 | 26782 | | |
| rs3786897 | *PEPD* | A | 0.552 | 1.10 | 78% | 11092 | 18436 | 55962 | | |
| rs1535500 | *KCNK16* | T | 0.368 | 1.08 | 60% | 11142 | 18520 | 56216 | | |
| rs16955379 | *CMIP* | T | 0.244 | 1.08 | 51% | 10771 | 17904 | 54345 | | |
| rs17797882 | *WWOX* | C | 0.792 | 1.08 | 42% | 66327 | 110247 | 334649 | | |

^a^Risk allele for type 2 diabetes reported in the previous reports

^b^Power estimation was performed using CaTS power calculator, CaTS: http://www.sph.umich.edu/csg/abecasis/CaTS/)

The prevalence of type 2 diabetes is assumed to be 10%, α = 0.05

^c^Required sample size estimation was performed using the Quanto software package (Version 1.2.4, http://hydra.usc.edu/gxe/).
