# Supplementary material for: Replication Study for the Association of 9 East Asian GWAS-Derived Loci with Susceptibility to Type 2 Diabetes in a Japanese Population
Source: PLoS One. 2013 Sep 25;8(9):e76317. doi: 10.1371/journal.pone.0076317 (PMC3783369; doi:10.1371/journal.pone.0076317)
Supplement: Table S4 — Power calculations were carried out using the Quanto software package (Version 1.2.4, http://hydra.usc.edu/gxe/). avalues are log-transformed for the analysis. (DOCX) [file pone.0076317.s004.docx]

**Table S4** Estimation of statistical power for the present study to detect associations of 9 SNPs with quantitative metabolic traits

|  | rs7041847 | | | rs6017317 | | | rs6467136 | | |
| --- | --- | --- | --- | --- | --- | --- | --- | --- | --- |
| ß | HOMA-IR^a^  (n=893) | HOMA-ß^a^  (n=893) | FPG^a^  (n=1,325) | HOMA-IR^a^  (n=893) | HOMA-ß^a^  (n=893) | FPG^a^  (n=1,325) | HOMA-IR^a^  (n=893) | HOMA-ß^a^  (n=893) | FPG^a^  (n=1,325) |
| 0.001 | 0.0502 | 0.0502 | 0.0584 | 0.0502 | 0.0502 | 0.0584 | 0.0501 | 0.0501 | 0.0561 |
| 0.005 | 0.0544 | 0.0543 | 0.2728 | 0.0543 | 0.0543 | 0.2706 | 0.0532 | 0.0531 | 0.2101 |
| 0.01 | 0.0677 | 0.0673 | 0.7737 | 0.0675 | 0.0671 | 0.7697 | 0.0627 | 0.0625 | 0.6343 |
| 0.03 | 0.2167 | 0.2129 | 0.9999 | 0.2150 | 0.2113 | 0.9999 | 0.1694 | 0.1667 | 0.9999 |
| 0.05 | 0.4989 | 0.4904 | 0.9999 | 0.4951 | 0.4866 | 0.9999 | 0.3833 | 0.3764 | 0.9999 |
| 0.1 | 0.9754 | 0.9728 | 0.9999 | 0.9743 | 0.9716 | 0.9999 | 0.9152 | 0.9093 | 0.9999 |
|  | rs831571 | | | rs9470794 | | | rs3786897 | | |
| ß | HOMA-IR^a^  (n=893) | HOMA-ß^a^  (n=893) | FPG^a^  (n=1,325) | HOMA-IR^a^  (n=893) | HOMA-ß^a^  (n=893) | FPG^a^  (n=1,325) | HOMA-IR^a^  (n=893) | HOMA-ß^a^  (n=893) | FPG^a^  (n=1,325) |
| 0.001 | 0.0502 | 0.0502 | 0.0578 | 0.0501 | 0.0501 | 0.0552 | 0.0502 | 0.0502 | 0.0584 |
| 0.005 | 0.0541 | 0.0540 | 0.2555 | 0.0527 | 0.0527 | 0.1873 | 0.0543 | 0.0543 | 0.2707 |
| 0.01 | 0.0663 | 0.0660 | 0.7403 | 0.0610 | 0.0607 | 0.5699 | 0.0675 | 0.0671 | 0.7699 |
| 0.03 | 0.2035 | 0.2001 | 0.9999 | 0.1523 | 0.1500 | 0.9999 | 0.2151 | 0.2114 | 0.9999 |
| 0.05 | 0.4682 | 0.4600 | 0.9999 | 0.3383 | 0.3322 | 0.9999 | 0.4953 | 0.4867 | 0.9999 |
| 0.1 | 0.965 | 0.9616 | 0.9999 | 0.8709 | 0.8636 | 0.9999 | 0.9743 | 0.9716 | 0.9999 |
|  | rs1535500 | | | rs16955379 | | | rs17797882 | | |
| ß | HOMA-IR^a^  (n=893) | HOMA-ß^a^  (n=893) | FPG^a^  (n=1,325) | HOMA-IR^a^  (n=893) | HOMA-ß^a^  (n=893) | FPG^a^  (n=1,325) | HOMA-IR^a^  (n=893) | HOMA-ß^a^  (n=893) | FPG^a^  (n=1,325) |
| 0.001 | 0.0502 | 0.502 | 0.0579 | 0.0501 | 0.0501 | 0.0562 | 0.0501 | 0.0501 | 0.0556 |
| 0.005 | 0.0541 | 0.0540 | 0.2574 | 0.0532 | 0.0532 | 0.2139 | 0.0529 | 0.0528 | 0.1960 |
| 0.01 | 0.0665 | 0.0661 | 0.7443 | 0.0630 | 0.0628 | 0.6442 | 0.0616 | 0.0614 | 0.5954 |
| 0.03 | 0.2050 | 0.2016 | 0.9999 | 0.1722 | 0.1695 | 0.9999 | 0.1588 | 0.1564 | 0.9999 |
| 0.05 | 0.4718 | 0.4635 | 0.9999 | 0.3906 | 0.3836 | 0.9999 | 0.3557 | 0.3492 | 0.9999 |
| 0.1 | 0.9663 | 0.9631 | 0.9999 | 0.9210 | 0.9154 | 0.9999 | 0.8898 | 0.8830 | 0.9999 |

Power calculations were carried out using the Quanto software package (Version 1.2.4, http://hydra.usc.edu/gxe/).

^a^values are log-transformed for the analysis
